# Building Health Equity Research Capacity in Myanmar Through a Virtual, Mentor-Based Training Program

**DOI:** 10.5334/aogh.5165

**Published:** 2026-08-18

**Authors:** Than-Tun Sein, Win Han Oo, Thin Mar Win, Ko Ko Zaw, Poe Poe Aung, Kyu Kyu Than, Robert Wachbroit, Phone Myint Win, Henry Silverman

**Affiliations:** 1 Department of Anthropology, Yangon University, Yangon, Myanmar; 2 Centre for Epidemiology and Biostatistics, Melbourne School of Population and Global Health, University of Melbourne, Australia; 3 Burnet Institute, Yangon, Myanmar; 4 STI Myanmar College, Yangon, Myanmar; 5 Malaria Consortium, Bangkok, Thailand; 6 University of Maryland School of Graduate Studies, USA; 7 University of Maryland School of Medicine, Baltimore, Maryland, USA

**Keywords:** health equity, research capacity strengthening, virtual training, fragile and conflict-affected settings, global health education, Myanmar

## Abstract

*Background:* In Myanmar, the COVID-19 pandemic and political unrest beginning in 2021 exacerbated longstanding health inequities and disrupted health services, universities, and research governance, rendering in-person research training infeasible when locally led health equity research was urgently needed.

*Objectives:* To describe the design, implementation, and evaluation of a fully virtual, mentor-based health equity research training program in Myanmar.

*Methods:* Supported by the Fogarty International Center/NIH, a one-year virtual training program integrated weekly virtual Zoom sessions, asynchronous learning through a learning management system, and structured faculty mentorship. Program evaluation employed a mixed-methods approach that included participant and faculty surveys, objective assessment of analytic projects using a standardized scoring rubric, and analysis of the program interpreted through the RE-AIM framework.

*Findings:* Of the initial 14 participants from civil society organizations and international nongovernmental organizations, 12 completed the program. Participants developed analytic projects using secondary datasets available through their organizations. These projects addressed health equity priorities, maternal health, COVID-19 impacts on informal workers, and disparities among displaced populations. Five subsequently produced full manuscripts. Objective evaluation demonstrated consistently high manuscript quality (mean rubric score 86.4/100, SD 2.8). Quantitative evaluations indicated high satisfaction with the program structure, learning resources, faculty engagement, and virtual mentoring. Qualitative findings highlighted the accessibility of the virtual format, gains in health equity knowledge and research skills, and the importance of sustained mentorship, while identifying the need for additional quantitative methods support and more individualized feedback.

*Conclusions:* A fully virtual, mentor-based training program can sustain health equity research capacity in fragile and conflict-affected settings when in-person training is not feasible. Beyond maintaining educational continuity, the program supported the development of high-quality analytic projects and scholarly manuscripts, demonstrating that context-sensitive virtual training can strengthen locally led research capacity and institutional resilience during prolonged humanitarian and political disruption.

## Introduction

Higher education has undergone a profound digital transformation, with universities increasingly leveraging virtual platforms to improve institutional efficiency and enhance learning experiences for students and faculty [1]. Hybrid models simultaneously deliver instruction to learners attending in person and remotely, integrating face-to-face teaching with real-time virtual engagement and shared digital resources to enhance flexibility and accessibility [2]. In contrast, a blended learning model integrates face-to-face teaching with online-mediated interactions (e.g., discussion forums) among students, educators, and learning resources [3]. In medical and public health education, blended learning has demonstrated value in supporting both theoretical knowledge acquisition and the development of clinical and analytical reasoning skills [4].

Distinct from hybrid and blended models, fully virtual approaches rely exclusively on online delivery and do not include in-person instructional components. Virtual approaches have increasingly enhanced health research capacity strengthening (HRCS) in low- and middle-income countries (LMICs), particularly where traditional in-person training models are limited by structural and resource constraints [5]. These constraints include unequal access to training opportunities, high costs, limited financing mechanisms, post-training workforce attrition, sustainability challenges, and limited rigorous program evaluation, which may mitigate selected barriers—most notably cost and geographic access—making them a viable alternative where in-person training is difficult to sustain [5].

These advantages are especially salient in settings where constraints are not incremental but systemic and prolonged, such as during political instability, armed conflict, or humanitarian crises [6, 7]. In these environments, educational institutions must maintain learning despite damaged infrastructure, displacement of faculty and students, and disrupted academic networks [8–11].

Recent experiences in Ukraine and Syria demonstrate how digital transformation, cloud technologies, learning management systems (LMSs), and virtual collaboration can preserve educational continuity and institutional resilience during war [8, 11, 12]. Additionally, reports from conflict-affected regions of northern Nigeria highlight how digital platforms can sustain educational access despite insecurity, school closures, and displacement [13]. Together, these experiences suggest that virtual learning environments can serve not only as emergency responses but also as mechanisms for maintaining academic capacity under conditions of prolonged disruption.

Myanmar, an LMIC in Southeast Asia, represents a similar context to other LMIC countries. The onset of the COVID pandemic combined with political unrest beginning in February 2021 exacerbated existing health inequities, disrupted health systems, and constrained higher education, disproportionately affecting ethnic minorities, internally displaced persons, and rural populations [12, 13]. At a time when research capacity was most needed to address these inequities, in-person training and mentorship became difficult to sustain.

In response to these constraints, our team began conceptualizing a fully virtual training model to support the continued participation of Myanmar-based scholars, community-based organization (CBO) representatives, and international partners in health equity research and research capacity strengthening. To our knowledge, no published studies have described a fully virtual, mentor-based research capacity-strengthening program specifically designed for implementation in fragile and conflict-affected settings.

This paper describes the structure, curriculum, delivery, and evaluation of a one-year fully virtual training program with particular emphasis on the strategies used to foster participant engagement, mentorship, and collaborative learning despite the substantial challenges posed by an adverse environment. In addition, it identifies key lessons learned that may inform the design, implementation, and adaptation of similar research capacity-strengthening initiatives in other LMICs facing conflict, political instability, or other adverse conditions.

## Methods

**Program Design and Educational Model:** The Health Equity Research Capacity Program in Myanmar was developed as a virtual learning initiative to strengthen the capacity of Myanmar-based academics and practitioners to conduct future research on the social and structural determinants of health, with an explicit focus on advancing health equity. The program was jointly coordinated by Myanmar academics and the faculty at the University of Maryland, Baltimore (UMB). It was designed to sustain research capacity building and academic collaboration during a period when in-person training was not feasible due to COVID-19–related movement restrictions and heightened political instability and security risks following the 2021 political unrest. The curriculum specifically focused on equipping participants with the theoretical, conceptual, and methodological foundations necessary to conduct rigorous health equity research [14, 15].

### Participants recruitment

Participants were recruited from Myanmar’s international nongovernmental organizations (INGOs), civil society organizations (CSOs), and international foundations involved in health-related initiatives. Recruitment utilized professional networks, emails, personal referrals, and word of mouth. The invitations outlined the training objectives, virtual format, anticipated time commitment, and opportunities for collaborative research development.

Selection was based on applicants’ professional roles, commitment from their mother organization to release the candidate for study and provide the necessary dataset, prior experience or interest in health equity research, written English skills, and capacity to engage in online learning despite inconsistent internet access. The process sought a balanced representation across disciplines and gender.

**Faculty Recruitment and Multidisciplinary Collaboration:** A multidisciplinary instructional team was convened to develop and deliver the curriculum, drawing on expertise in public health, epidemiology, medicine, bioethics, biostatistics, qualitative methods, and community engagement. The faculty included senior scholars from Myanmar and the UMB, with extensive experience in strengthening research capacity in low- and middle-income contexts. This integrative pedagogical model reflects a deliberate effort to provide participants with a comprehensive and contextually grounded learning experience [16]. The faculty composition exposed trainees to both local and international perspectives on health equity, encompassing theoretical frameworks, methodological approaches, ethical considerations, and community-engaged research practices. In addition, close collaboration among faculty members supported curricular coherence across modules by aligning the conceptual content with technical skills and applied competencies.

**Faculty Development:** Faculty members received training in virtual pedagogical strategies, emphasizing interactive engagement and effective feedback to support learning in a remote setting [17]. Faculty preparation also included structured orientation to the Moodle LMS to ensure proficiency with virtual instructional tools and consistent course delivery [18]. Together, these preparatory activities supported effective teaching and mentoring in resource-constrained and politically unstable LMIC contexts, aligning with established remote-learning models [19–21].

**Curriculum Design:** The curriculum was developed collaboratively by our ensembled multidisciplinary faculty team with input from international advisors. The content focused on building competencies in health equity research methods and applying ethical principles. The key thematic areas included the following:

- Foundations of Health Equity Research: Concepts and frameworks of health equity, social justice, social determinants of health.
- Identifying and prioritizing inequities within local contexts.
- Developing the research question and identifying the research gap.
- Research design for equity-focused studies (quantitative including biostatistics, qualitative, mixed methods, implementation research, systematic review and meta-analysis, secondary data analysis).
- Community engagement and participatory research approaches.
- Research ethics in fragile contexts.
- Principles of scientific writing and preparing a manuscript for peer review.

**Delivery Format and Pedagogical Approach:** The program was delivered over one year using a blended virtual format that combined synchronous webinars with asynchronous, online activities. This structure enabled sustained engagement while accommodating participants’ variable internet connectivity and professional responsibilities. The pedagogical approach emphasizes interactive learning through small group work, collaborative problem solving, and participant-led presentations.

**Virtual Webinars:** Weekly synchronous sessions were conducted on the Zoom platform. Each session lasted 2 hours and included a 45-minute faculty presentation, followed by approximately 15 minutes of questions and answers. For the subsequent hour, participants engaged in breakout group discussions regarding a case study, which was followed by a facilitated large-group discussion.

**Moodle LMS:** Participants received training on the use of the Moodle LMS during program orientation. Moodle served as the central repository for program materials and supported asynchronous learning activities, including assigned readings, discussion board participation, and short applied assignments. Homework requirements were adapted as needed to accommodate the participants’ competing professional responsibilities and personal constraints, including family health obligations.

**Virtual Mentoring:** Virtual mentoring was integrated into the program as a core structural component. Each participant was paired with a primary faculty mentor and a secondary co-mentor from the participant’s home organization to provide academic guidance and institutional context. Mentors supported participants in engaging with didactic content and developing and refining proposals focused on locally relevant health equity challenges.

Mentoring expectations were established at the program’s outset, while allowing mentor–mentee pairs to adjust communication frequency, mode of interaction, and areas of focus over time to accommodate professional responsibilities and evolving project needs [22]. This flexibility was particularly important given the current context in Myanmar, which frequently disrupted synchronous engagement and necessitated asynchronous communication and alternative mentoring strategies to ensure continuity [23]. Mentor assignments were based on the alignment of expertise, project relevance, and availability rather than gender matching, an approach consistent with effective virtual capacity-building models [17].

**Capstone Analytic Projects:** Participants conducted analytic projects using de-identified secondary data drawn from datasets maintained by their institutions or from previously completed studies. These projects were undertaken as educational capstone exercises designed to develop competencies in research design, data analysis, interpretation, and scientific communication. The projects involved no collection of new data, no intervention or interaction with human participants, and no access to identifiable private information.

### Evaluation methods

**Evaluation of Participants’ Performance:** Participants’ performance was assessed using formative and summative evaluation approaches. Formative evaluation consisted of informal mentor–mentee debriefing sessions conducted throughout the program. These sessions provided ongoing feedback from participants and enabled faculty to make iterative adjustments to content delivery, instructional pacing, and technical support.

**Summative Evaluation:** Summative evaluation was based on a performance assessment of participants’ successful completion of their analytic projects. Two of the faculty members independently evaluated each manuscript using a standardized scoring rubric that assessed key elements of proposal development, including the significance of the research topic, quality of the literature review, identification of the research gap and formulation of the research question, clarity of the study objectives, appropriateness of the study design, the extent to which the project addressed a health equity issue, and overall quality of the writing. The scoring rubric is provided in **Supplementary File S1**. These structured assessments provided an objective measure of participants’ ability to synthesize and apply the knowledge and skills acquired throughout the program.

**Rationale for RE-AIM:** While RE-AIM was initially developed to evaluate public health interventions [24], its domains are directly applicable to educational and capacity-building programs in resource-constrained settings, where issues of accessibility, feasibility, adoption, and sustainability are as critical as educational outcomes. (See **Supplementary File S2** for Conceptual Mapping: Educational Programs and RE-AIM.) Application of the RE-AIM framework to evaluate this capacity-building program resonates with prior applications of RE-AIM to workforce training initiatives, demonstrating the framework’s utility beyond traditional public health interventions and in training program evaluation [25–27]. Consistent with prior applications of RE-AIM in educational and workforce training initiatives, this approach was intended to support the systematic interpretation of qualitative and descriptive quantitative data, rather than to generate causal inferences or comparative implementation outcomes.

**Participant Evaluation of the Program:** At the conclusion of the program, participants completed an anonymous online survey administered through SurveyMonkey. The survey included multiple-choice and open-ended questions that assessed participants’ self-perceived gains in knowledge, research skills, and confidence in designing and conducting health equity research. Participants also evaluated the overall program by identifying its strengths and providing suggestions for improvement.

**Faculty Evaluation of the Program:** Faculty members completed a separate survey consisting of multiple-choice and open-ended questions that evaluated their instructional experience, the feasibility of the fully virtual program format, the mentoring workload, and the perceived strengths, challenges, and opportunities for improving program implementation.

**Analysis of the End of Program Surveys:** We conducted a mixed-methods evaluation of the student and faculty surveys.

**Quantitative Data Analysis:** The survey items employed a five-point Likert scale ranging from 1 = strongly agree to 5 = strongly disagree. The items assessed participants’ perceptions of key program components, including course structure, content, workload, and the usability of the Moodle LMS, as well as their evaluations of instructional quality. Quantitative survey data were analyzed using descriptive statistics to summarize participants’ responses to the Likert-scale items. Frequencies and percentages were calculated to characterize the overall perceptions of program structure, content, workload, learning platforms, and instructional quality. Where appropriate, the responses were aggregated to facilitate interpretation and reporting.

**Qualitative Analysis:** Open-ended responses were collected from three survey questions: “What were the strengths of the program?” “How could the program be improved?” and “What was your experience developing your analytic project?” All qualitative data were uploaded into MAXQDA (Version 2024) for data management and organization [28]. Qualitative data from the end-of-program student evaluation survey were analyzed using thematic analysis with a combined inductive–deductive approach. Inductive coding was used to identify the themes emerging directly from the participants’ narratives. Data were coded deductively using the domains of the RE-AIM framework (Reach, Effectiveness, Adoption, Implementation, Maintenance) to guide the evaluation of the capacity-building program, while codes were iteratively refined and organized into higher-order themes corresponding to each RE-AIM domain. This analytic approach enabled a systematic assessment of online accessibility and compatibility with full-time employment (Reach), knowledge gains in health equity and research skill development (Effectiveness), faculty expertise and mentoring engagement (Adoption), need for enhanced feedback and quantitative support (Implementation), and desire for credentialing, credit-bearing structure, and continued platform access (Maintenance).

**Ethical Considerations:** The Health Equity Research Capacity Program was established as an educational and research capacity-strengthening initiative. The primary purpose of the educational program was to develop, deliver, and continuously improve a virtual training program that enabled Myanmar health professionals to acquire competencies in health equity research. The educational activities described here were conducted in the same manner as routine instructional practices used in graduate research training programs, where curriculum development, student learning exercises, and program evaluation are undertaken to improve education rather than to conduct research.

Similarly, the participants’ analytic projects were educational capstone exercises intended to develop competencies in research design, data analysis, interpretation of findings, and scientific communication. Participants analyzed de-identified secondary datasets to which they already had legitimate institutional access through their employing organizations. The projects involved no collection of new data, no intervention or interaction with human participants, and no access to identifiable private information. Their sole purpose was educational—to develop competencies in research design, data analysis, interpretation, and scientific communication through analysis of existing de-identified data.

Finally, consistent with routine educational practice, participant and faculty feedback was collected to evaluate the curriculum, mentoring model, and virtual learning environment and to identify opportunities for program improvement. Participation in the program evaluation surveys was voluntary and anonymous, and survey findings are reported only in aggregate to protect participant privacy. These educational evaluation activities were undertaken as program assessment and quality improvement rather than as a prospective research study designed to answer scientific questions beyond the educational objectives of the program.

Taken together, the educational program, participants’ analytic projects, and program evaluation activities were all implemented to support instruction, competency development, and continuous program improvement.

## Results: Program Outcomes

### Participant characteristics

Fourteen participants were enrolled in the Virtual Health Equity Program. Of these, 12 (86%) completed the 1-year program while 2 students exited the program due to family and personal health issues. The cohort reflected gender diversity and was predominantly composed of individuals from CSOs andINGOs. The complete demographic characteristics are presented in Table 1.

**Table 1:** Demographic characteristics of health equity participants (n = 14).

CHARACTERISTICS	N	%
Gender		
Female	9	64%
Male	5	36%
Education attainment (highest degree)		
MD + MPH	3	21%
MD	3	21%
MPH	3	21%
MBBS	3	21%
BS	2	14%
Affiliation		
International non-governmental organization (INGO)	10	72%
Civil society organization	2	14%
Research foundation	2	14%
Age (years)		
Mean	32.5	
SD	4.64	

### Academic program

The academic program was delivered over the 2022–2023 calendar year and followed a structured curriculum that progressed from foundational concepts in health equity and social determinants of health to research design, secondary data analysis, quantitative and qualitative methods, and scientific and grant writing skills. The program also included dedicated modules on community-based participatory research, research ethics, and responsible scholarly practices, with multiple opportunities for participants to present and receive mentored feedback. The full academic calendar and session sequences are presented in Table 2.

Table 2Health equity research—agenda (2022–2023).

**Table:** **1. Foundations of health equity research**: This module/workshop will introduce the core principles of health equity research and cover topics such as defining health equity, social determinants of health, cultural competence, and examples of health equity research.

#	DATE	TOPIC	LEARNING OBJECTIVES
1.1	October 10, 2022	Introduction to concept of health equity	Define health equity Explain the core principles of health equity Distinguish between equality and equity Discuss the importance of cultural competence to address health inequities
1.2	October 17, 2022	Social justice and ethical frameworks for health equity research	Identify how particular theories of justice underlie the idea of health inequity Discuss the special importance addressing health inequities for the health professional Analyze different theories of justice that can serve as a justice framework for international clinical research
1.3	October 24, 2022	Social determinants of health: From health disparities to health equity	Define political and social determinants of health (SDH) Discuss conceptual framework of SDH Describe key dimensions and directions for policy to address SDH Discuss the importance of social cohesion in addressing SDH Discuss racial/ethnic inequalities as determinants of health inequities
1.4	October 31, 2022	Health equity research: Types of research that addresses health equity	Identify the methodologies important in equity research Explain community-based participatory research Discuss mixed-methods research Discuss of ethics of equity research

**Table:** **2. Developing the research question for choosing a proposal**:

#	DATE	TOPIC	LEARNING OBJECTIVES
2.1	November 7, 2022	Developing the research question	Define the research gap Determine the research gap

**Table:** **3. Research methods and students’ presentations on research questions/proposal and data availability**: This module will help students to design their research and collect, analyze, and interpret information. The research methods are the techniques used for performing different activities during the research projects. The selection of an appropriate research method is extremely important as it has a significant influence on the research outcome. Participants will choose a research topic during the first few months of the Health Research Equity training followed by the write-up of a proposal. Participants are encouraged to conduct secondary analysis of an existing database. Each participant will be guided by a mentor and will also receive help from a statistician.

#	DATE	TOPIC	LEARNING OBJECTIVES
3.1	November 12, 2022	Literature searching	Explain literature review and systematic review, and their process in exploring health equity Search in online databases for health equity review questions using the Boolean or MeSH search query Retrieve and manage literature
3.2	November 21, 2022	Secondary data analysis	Explain key concepts directly applicable in data analysis for health equity research Develop a statistical analysis plan for participants’ own research projects
3.3	November 28, 2022	Systemic reviews and meta-analysis	Perform systematic reviews
3.4	December 5, 2022	Practical session for literature search	Perform a literature review exercise
3.5	December 12, 2022	Quantitative research	Describe quantitative health research Discuss the role of quantitative research in evaluating health equity List different types of quantitative research (epidemiological study designs) in evaluating health equity Discuss pros and cons of each epidemiological study designs in health equity research
3.6	December 19, 2022	Research objectives	Explain how to develop research objectives
3.7	January 9, 2023	Students’ presentation	Presentation of summary research questions, objectives, and data availability
3.8	January 16, 2023	Students’ presentation	Presentation of summary research questions, objectives, and data availability
3.9	January 23, 2023	Qualitative research	Describe qualitative health research for health equity Explain the different methodologies/theories associated with qualitative research Explain the process of designing qualitative health equity research List different types of qualitative data collection methods in evaluating health equity Identify the four types of qualitative data analysis including characteristics of each data analysis method Describe how to apply rigor in qualitative health research
3.10	January 30, 2023	Statistical methods I	Identify the steps in data management to produce clean and tidy data for data analysis and Carry out key data management tasks using SPSS
3.11	February 6, 2023	Statistical methods II	Describe key concepts directly applicable in data analysis for health equity research Develop a statistical analysis plan for participants’ own research projects
3.12	February 13, 2023	Statistical methods III	Produce descriptive statistics and perform main statistical analyses using SPSS Interpret and transform SPSS outputs to the tables of the result session of the report and Interpret the tables in the result session in relations to health equity analysis
3.13	February 20, 2023	Research in the digital world	Explain social and digital methods to conduct research

**Table:** **4. Scientific and grant writing part I**: This workshop will review best practices in scientific writing style, analyze the main genres of research writing, provide many opportunities for participants to revise their own work based on feedback from instructors and each other.

#	DATE	TOPIC	LEARNING OBJECTIVES
4.1	February 27, 2023	The research paper (IMRaD format)	Describe the “language” of scientific writing
4.2	March 6, 2023	Introduction section	Write a succinct introduction section that describes the research gap

**Table:** **5. Best practices in engaging communities in research**: Encouraging and supporting community participation in generating evidence for improved solutions to health challenges are important in building trust in science through sustained engagement. As such, there is a need for the promotion of community engagement in health research. Community participation needs to occur at all levels of the research process, including framing the research questions and participation in the development of the study design. This is expected to improve the uptake and implementation of research findings.

#	DATE	TOPIC	LEARNING OBJECTIVES
5.1	March 13, 2023	Theory of community-based participatory research and methods	Describe historical and practice roots of CBPR Discuss Socio-economic framework for CBPR Define the community
5.2	March 20, 2023	Best practices in engaging communities	Describe partnership formation and maintenance Explain Power, trust, and dialogue Describe working with diverse communities Use stakeholder theory to engage stakeholders
	March 27, 2023		Public holiday and no lecture
5.3	April 3, 2023	Types of implementation strategies	Describe implementation research (IR) and its key characteristics Contextualize the IR issues Design and plan a IR research Discuss best practices for engaging community in rolling out of an IR

**Table:** **6. Scientific and grant writing part II**:

#	DATE	TOPIC	LEARNING OBJECTIVES
6.1	April 10, 2023	Methods and results sections	Describe and justify methodological choices Report results accurately and transparently
6.2	April 17, 2023	Discussion section	Interpret key findings in relation to study objectives and existing literature Critically assess study limitations and strengths Articulate the broader implications of the findings
6.3	April 24, 2023	Plagiarism and how to avoid plagiarism	Explain plagiarism Describe the factors that lead to plagiarism Explain methods on how to avoid plagiarism
6.4	May 1, 2023	Submitting a manuscript for peer review	Prepare a manuscript for submission by correctly formatting all required components Identify and apply journal selection criteria Demonstrate effective engagement with the peer-review process
6.5	May 8, 2023	Citing sources	Demonstrate how to cite sources in your manuscript

### Capstone analytic projects

Each participant or team developed an analytic project addressing a locally relevant health equity issue through secondary data analysis. The faculty mentors guided the participants through each stage of their project development, including developing analytic plans for existing datasets. The proposals covered a range of topics, including gender inequities in health access, nutrition disparities among displaced populations, the impact of COVID-19 on informal workers, prevention of malaria among mobile migrant populations, and inequities in maternal health outcomes. Other projects explored mental health challenges among conflict-affected communities, inequitable access to vaccination, and the relationship between poverty and chronic disease management. Reflecting the program’s emphasis on collaboration and applied learning, three participants worked jointly on a single proposal, resulting in 10 distinct research projects (see Table 3).

**Table 3:** List of capstone projects.

NO.	TITLE	THEMATIC AREA
**1**	Geographic disparities of private clinical laboratories in Yangon	Laboratory assessment
**2**	Factors affecting on depression and anxiety, and its social determinants during the time of conflict, Kayin State, Myanmar	Mental health
**3**	Socioeconomic status influencing the complementary feeding practices among children aged 6–23 months in Kayin State	Nutrition
**4**	Inequity in HIV index testing, treatment and prevention services in The Union Project	HIV
**5**	Malaria incidence and access to the personal protective package among the forest goers in forest fringe villages of Kachin State, Myanmar: Quasi-experimental study	Malaria
**6**	Treatment success rate of HCV treatment and its associated factors among HIV/HCV co-infected people who inject drugs (PWID) from three MDM project sites—Myitkyina, Moegaung, and Hopin, Kachin State, Myanmar	Hep. C
**7**	Institutional delivery and its determinants in eight essential health project townships in Myanmar	MRH
**8**	Anti-retroviral therapy (ART) coverage among people living with HIV/AIDS (PLHIV) from an NGO-based facility	HIV
**9**	Assessing the association of the social determinants with the low dietary diversity among women reproductive age (WRA) and 6–23 months old children in the rural district of Puta-O (northernmost part of), Myanmar	Nutrition
**10**	Accessibility of improved water, sanitation, and hygiene (WASH) facilities and its associated factors in rural area of Hlaingbwe township, Kayin Sate	WASH

### Evaluation of the training program

**Quantitative Findings:** All participants accessed the end-of-program feedback survey, with 11 providing complete responses to the survey. The results showed a high level of participant satisfaction with the virtual training model, course design, and faculty engagement.

Overall, 82% of respondents (combining “agree” and “strongly agree”) reported that the course was well-structured and effective in supporting their understanding of the core principles of health equity. Most participants (91%) agreed that the learning resources were helpful, and 82% found the workload to be appropriate for the course level. In addition, 90% reported that the online learning platform was easy to navigate, reflecting their successful adaptation to the virtual learning environment. Furthermore, 91% of the students indicated that the course met or exceeded their expectations. Faculty performance was also rated highly: all respondents (100%) agreed or strongly agreed that instructors were well-prepared and communicated expectations clearly, while 91% reported that faculty encouraged active participation and provided helpful feedback on assignments and analytic projects. All the respondents indicated that they would recommend the course to others. Taken together, these findings suggest strong participant engagement and satisfaction despite the challenges associated with distance learning, political instability, and competing professional responsibilities of the participants.

### Qualitative findings organized by RE-AIM

Thematic analysis of open-ended survey responses, structured using the RE-AIM framework, identified consistent strengths and areas for improvement across domains. (**See Supplementary File S3**)

**Reach:** Online Accessibility and Compatibility with Full-Time Employment: Participants emphasized that the virtual format and outside office hours scheduling enabled their participation. As one student noted, “*Online platform is the strength point because everyone can join*.” Another noted that the flexibility of scheduling allowed professionals balancing employment responsibilities to participate fully: “*Online learning and learning with a full-time job is also a strength of this course… the schedule is one of the strong points*.”

**Effectiveness:** Knowledge Gains in Health Equity and Research Skill Development: Students reported meaningful gains in health equity knowledge and research competencies, particularly in secondary data analysis. One participant reflected, *“I understand more about health equity professionally and the process of conducting research on secondary data properly.”* Another participant described, *“The students in Myanmar can have the opportunity to study health equity fundamentals and issues from prominent and highly skilled teachers…”* Many students described the project process as transformative, with one writing, “*Despite the struggles, each hurdle provided valuable learning experiences, contributing to the overall excellence of the project*.”

**Adoption:** Faculty Expertise and Mentoring Engagement: Faculty expertise and mentoring engagement are cited as key contributors to program quality and credibility. As one student stated, “*Teachers involved in the course have much experience on research and health equity issues*.” Others highlighted supportive mentorship as critical to their success: “*The faculty, research supervisor, and my organization supported the research project… heartfelt thanks to all the teachers for providing this learning opportunity*.”

**Implementation. Need for Enhanced Feedback and Quantitative Support:** Although overall program delivery was viewed positively, participants identified opportunities to strengthen implementation through more intensive feedback and additional support for quantitative methods. One participant remarked, *“Trying to understand the statistics and SPSS online alone is hard for me.”* Many participants expressed a desire for more individualized feedback sessions to review their work, address methodological questions, and receive tailored guidance. As one participant noted, *“Individual review and feedback sessions may be required for the students,”* adding that structured follow-up opportunities, such as periodic in-person meetings or quarterly learning sessions, would further strengthen learning outcomes. Collectively, these comments highlighted the importance of more interactive and sustained mentoring, particularly to reinforce quantitative skills and facilitate the application of newly acquired research competencies over time.

Beyond these educational needs, program implementation also required continual adaptation to an evolving humanitarian and political context. Participants experienced intermittent internet disruptions, electricity outages, relocation, competing humanitarian responsibilities, and heightened concerns regarding personal security. To maintain continuity, faculty adopted flexible scheduling, asynchronous learning opportunities, recorded educational materials, individualized mentoring, and modified assignment deadlines when necessary. Because of ongoing security considerations, some contextual adaptations cannot be described in greater detail; however, these experiences underscore the importance of flexibility, trust, and sustained mentorship for implementing research capacity-strengthening programs in fragile and conflict-affected settings.

**Maintenance:** Desire for Credentialing, Credit-Bearing Structure, and Continued Platform Access: Participants emphasized the importance of formalization and long-term value, including credit-bearing structures and enhanced certification. As one student suggested, *“If this program could be set up like a diploma program with a credit system, this may be more rewarding and beneficial.”*

### Faculty evaluation of the program

Faculty feedback provided complementary perspectives on the feasibility, instructional demands, and sustainability of the virtual training model. Six faculty members completed a post-program survey that included quantitative ratings and open-ended responses (**see Supplementary File S4**).

**Quantitative Findings:** Results indicated strong faculty endorsement of the program’s instructional structure and value. All faculty agreed or strongly agreed that the weekly virtual sessions were effective for teaching course content. A total of 83% of faculty endorsed the virtual mentoring component as adding meaningful value to the program and indicated that they would recommend participation to colleagues, suggesting favorable faculty adoption despite the challenging political and institutional context.

Faculty responses were more mixed regarding student engagement with asynchronous learning activities and the adequacy of technical support for the Moodle LMS. While 83% strongly agreed or agreed that asynchronous materials complemented the live webinars effectively, there was variability in the faculty responses regarding the extent of student engagement with the asynchronous activities. For example; four of six faculty strongly agreed or agreed that the students engaged meaningful with the asynchronous materials, whereas one faculty was uncertain and one disagreed.

**Qualitative Findings:** Feedback from the faculty underscored the value of sustained mentorship in enabling students to engage with the program. When asked to comment on the value of mentoring, one faculty member said: “*communications with the students*,” and another noted that “*participants more open discussed their struggles and their needs during virtual mentoring than during lecture time*.” At the same time, faculty emphasized the importance of greater structure in mentoring. For example, one faculty remarked that “*its integration and execution could be strengthened to deepen its impact…. I suggest providing mentors with brief orientation materials or best-practice guidelines for virtual mentoring, particularly in an online environment, could enhance the quality and consistency of mentoring.”* Most made recommendations for future iterations of the program. For example, one respondent recommended, “*Moodle support for trainers and students,”* another faculty said:* “Improve alignment between asynchronous activities and weekly virtual sessions by explicitly referencing forum discussions or assignments during live webinars, which may encourage greater student participation*.” Another recommended “*Address student readiness for virtual learning by requiring a short, mandatory pre-course module on ‘How to Learn Online,’ as many students appeared unprepared to engage meaningfully in a fully virtual format*.” Finally, one faculty advocated for “*less assignments for the students*.”

**Objective Assessment of Scholarly Analytic Projects:** Twelve participants completed their analytic proposals of which three worked on one proposal together. All completed the didactic curriculum and developed their proposals. Five participants **(50%)** submitted finished manuscripts, while the remaining seven were unable to finalize a manuscript during the program period due to a variety of reasons that included substantial professional demands placed on participants, the challenging operational environment in Myanmar during the study period, and personal or family health reasons.

Individual rubric scores for each manuscript are shown in Table 4. Among the five completed manuscripts, the mean total rubric score was 86.4 out of 100 (SD 2.8; range 83–89.5), indicating consistently strong performance across all participants. To facilitate comparisons across rubric domains with different maximum point values, mean scores were calculated as a percentage of the maximum possible score for each domain. Relative performance was highest for Research Objectives and Research Question (93.3%) and lowest for Potential Impact and Health Equity and Organization and Writing Quality (both 84.0%).

**Table 4:** Rubric scores of the completed manuscripts (n = 5).

MANUSCRIPT TITLE	IMPORTANCE AND SIGNIFICANCE (20)	LITERATURE REVIEW AND JUSTIFICATION (15)	RESEARCH OBJECTIVES AND RESEARCH QUESTION (15)	METHODS AND SCIENTIFIC RIGOR (20)	POTENTIAL IMPACT AND HEALTH EQUITY (20)	ORGANIZATION AND WRITING QUALITY (10)	TOTAL (100)
Association between distribution of personal protection package to malaria at-risk populations and accessibility to malaria services	16.5	12.5	13.5	16	17	8.5	84.0
Determinants of birth delivery	17.5	13	14	17.5	16.5	8.5	87.0
Association of the social determinants with dietary diversity	17.5	13.5	14.5	17.5	17.5	9	89.5
Factors with HCV treatment success	18	13.5	14.5	18	16.5	8	88.5
Geographic disparities of private clinical laboratories	16.5	12.5	13.5	16	16.5	8	83.0
Mean (SD)	17.2 (0.7)	13.0 (0.5)	14.0 (0.5)	17.0 (0.9)	16.8 (0.4)	8.4 (0.4)	86.4 (2.8)

## Discussion

Guided by the RE-AIM framework, this study evaluated the implementation and impact of a fully virtual, mentor-based health equity research training program conducted in a fragile, politically constrained setting. Using RE-AIM as an interpretive framework allowed us to examine not only educational outcomes but also the reach, adoption, implementation, and sustainability of a research capacity-strengthening intervention delivered amid institutional disruption.

Across RE-AIM domains, the findings demonstrate that health equity research capacity can be strengthened even when in-person training is not feasible. Rather than emphasizing participant outcomes alone, the evaluation highlights how virtual accessibility, sustained mentorship, and the applied use of secondary data enabled continued engagement and skill development despite mobility restrictions, security concerns, and infrastructure challenges. The convergence of student and faculty perspectives across RE-AIM domains—particularly regarding program reach, instructional effectiveness, adoption of the virtual model, and implementation challenges—further strengthens the credibility of these findings.

Several program features appeared to contribute to the program’s feasibility and acceptability. A multidisciplinary faculty provided complementary expertise in public health, ethics, quantitative and qualitative methods, and community engagement, reflecting the interdisciplinary nature of health equity research. Longitudinal mentoring, integrated throughout proposal development rather than limited to the post-course period, enabled participants to immediately apply course concepts, receive iterative feedback, and refine their projects as the curriculum progressed. Regular mentor–mentee interactions also promoted continuity, experiential learning, and professional support despite geographic dispersion. At the same time, qualitative feedback identified opportunities to strengthen implementation through more individualized mentoring and additional support for quantitative methods in the fully virtual environment.

The RE-AIM framework also highlighted factors influencing program implementation. Viewed through the lens of adoption, sustained faculty engagement and mentors’ willingness to recommend future iterations suggest that the virtual mentor-based model was both feasible and acceptable from the instructors’ perspective. However, implementation was challenged by unreliable internet connectivity, coordination across time zones, and the difficulty of teaching statistical software remotely. These educational barriers were compounded by the broader political and humanitarian crisis, which affected both participants and faculty, reinforcing observations from prior studies of virtual education in conflict-affected settings and underscoring the importance of flexibility, psychosocial support, and sustained mentoring [29, 30].

Finally, the maintenance dimension emphasized that sustaining the program’s impact will require institutionalization through credentialing, integration into credit-bearing curricula, and continued access to learning platforms beyond the initial funding period. Together, these findings suggest that virtual mentor-based training represents a practical and scalable strategy for strengthening research capacity in fragile settings, if implementation is accompanied by sustained mentoring, institutional support, and strategies to address the educational and contextual challenges inherent to conflict-affected environments. This interpretation is consistent with previous RE-AIM–based evaluations of educational and workforce development initiatives that distinguish program reach, adoption, implementation, and long-term sustainability [25].

### Faculty teaching experience and mentoring

From the faculty perspective, delivering the program required substantial pedagogical adaptation and sustained cross-institutional collaboration. Preparation in online teaching strategies and effective use of the LMS were essential for maintaining engagement and instructional continuity. Although the mentor-based model enabled individualized guidance, it required considerable faculty time, particularly during proposal development and data analysis. These experiences are consistent with previous reports highlighting both the benefits and demands of virtual mentoring in resource-constrained settings [5, 31, 32].

Although the curriculum was grounded in internationally recognized frameworks of health equity and the social determinants of health, participants were encouraged to apply these frameworks to Myanmar-specific circumstances, including ethnic and geographic disparities, displacement, political instability, and unequal access to health services. The program did not, however, systematically elicit participants’ or communities’ own definitions of health equity, justice, or social responsibility. Future iterations could address this limitation through participant-led discussions and community-engaged activities that identify locally grounded understandings of fairness, collective responsibility, resilience, and equitable access to health resources. Such perspectives could complement international frameworks and further strengthen the contextual relevance of the curriculum.

### Capstone analytic projects

Regarding the capstone projects, all participants successfully completed a research proposal. Notably, five participants progressed beyond proposal development to produce final manuscripts addressing diverse health equity challenges in Myanmar, including malaria prevention, hepatitis C treatment, maternal health, nutrition, and access to clinical laboratory services. Producing a complete manuscript represents a substantial scholarly achievement, requiring participants to conduct data analyses, interpret findings, and communicate results in a format suitable for scientific dissemination. The scores among these completed manuscripts suggest that participants were strongest in formulating clear research questions and objectives. The relatively lower scores for Potential Impact & Health Equity and Organization & Writing Quality indicate areas where additional mentoring could strengthen future cohorts, particularly in articulating the broader significance of the work and improving scientific writing.

Given the profound political instability, armed conflict, disruptions to health services, and competing professional responsibilities experienced throughout the program, this level of scholarly productivity provides compelling evidence that a fully virtual, mentor-based training model can foster meaningful research capacity strengthening in fragile and conflict-affected settings.

At the same time, the inability of several participants to progress from research proposals to completed manuscripts reflected the extraordinary external circumstances that limited their ability to devote sustained time to research. Rather than indicating shortcomings of the program itself, these challenges underscore the need for capacity-strengthening initiatives in fragile and conflict-affected settings to anticipate such barriers and provide additional support to maximize scholarly productivity.

Future iterations of the program could incorporate structured manuscript-writing workshops, protected writing time, and continued post-program mentoring to facilitate manuscript completion and publication. Extending the evaluation beyond program completion will also be important to assess longer-term outcomes, including peer-reviewed publications, successful grant applications, career advancement, and leadership roles in health equity research over the subsequent 3–5 years.

**Effects of environmental instability on program implementation.** Program implementation required continual adaptation to rapidly changing circumstances. Participants experienced intermittent internet disruptions, electricity outages, relocation, competing humanitarian responsibilities, and heightened concerns regarding personal safety. Faculty responded by adopting flexible scheduling, asynchronous learning, recorded lectures, individualized mentoring, and modified assignment deadlines. Although security considerations preclude describing some contextual adaptations in greater detail, these experiences underscore the importance of flexibility, trust, and sustained mentorship when implementing research capacity-strengthening programs in fragile and conflict-affected settings.

### Implications for research capacity building in fragile contexts

Our findings complement reports describing digital education initiatives in other fragile and conflict-affected settings while highlighting important differences in educational objectives [8, 29, 33].

For example, studies from Ukraine have emphasized how digital transformation enabled universities displaced by war to preserve institutional continuity through LMSs, cloud-based technologies, virtual classrooms, and sustained communication despite widespread disruption [8, 11]. Similarly, a report from northern Nigeria described how digital platforms maintained educational access during prolonged insecurity and school closures by providing flexible online learning opportunities where conventional classroom instruction was no longer feasible [33].

Like these initiatives, our program demonstrated that virtual platforms could preserve educational continuity despite conflict-related disruption, geographic dispersion, and infrastructure challenges. However, unlike programs primarily designed to sustain undergraduate or university education, the Myanmar initiative focused specifically on developing health equity research capacity through longitudinal mentorship integrated with experiential proposal development. Rather than simply maintaining educational access, the program sought to cultivate locally led research capable of generating evidence to address health inequities exacerbated by political instability and conflict. These findings suggest that virtual learning platforms can serve not only to sustain education during crises but also to strengthen research capacity that supports long-term recovery and health system resilience.

### Future directions: implications for implementation and maintenance

Future iterations should strengthen both implementation and long-term sustainability. Priorities include expanding quantitative methods instruction, providing structured manuscript-writing support and protected writing time, increasing opportunities for individualized mentoring, and adopting tiered mentoring models to improve faculty sustainability. As security conditions permit, selective hybrid elements, formal academic credentialing, and stronger South-South collaboration could further enhance program impact. Given the unique psychosocial burdens associated with conducting research during conflict, future programs should also integrate peer-support mechanisms and access to mental health resources. Finally, longitudinal evaluation is needed to determine whether these educational gains translate into sustained research productivity, career advancement, and leadership in health equity research.

### Limitations

This study has several limitations. First, qualitative findings were based on participant and faculty self-reports and therefore reflect perceived experiences rather than objective measures of competence or program impact. Second, the RE-AIM framework was used as an interpretive framework rather than a formal implementation evaluation instrument; consequently, findings related to RE-AIM domains should be interpreted as perceptions of program reach, adoption, implementation, and maintenance rather than objective measures of effectiveness. Finally, the evaluation focused on immediate educational and implementation outcomes and did not assess longer-term indicators of research capacity strengthening, such as publications, grant funding, career advancement, or leadership in health equity research.

### Conclusion and broader significance

This study adds to the growing evidence that virtual, mentor-based training can strengthen health research capacity in LMICs when political instability and conflict preclude conventional in-person education. Beyond developing research skills, the program fostered sustained mentorship, scholarly engagement, and locally relevant research addressing pressing health equity challenges. These findings suggest that context-sensitive virtual training programs can serve not only as pragmatic responses to educational disruption but also as sustainable strategies for strengthening locally led research capacity and advancing health equity in fragile and conflict-affected settings.

## Additional Files

The additional files for this article can be found as follows:

Supplementary File S1. Conceptual Mapping: Educational Programs and RE-AIM.

Supplementary File S1. Student Feedback Mapped to RE-AIM Domains.

Supplementary File S3. Faculty Feedback Mapped to RE-AIM Domains.

Supplementary File S4. Myanmar Health Equity Proposal Evaluation Rubric.
