# Supplementary material for: Building Health Equity Research Capacity in Myanmar Through a Virtual, Mentor-Based Training Program
Source: Ann Glob Health. 2026 Aug 18;92(1):80. doi: 10.5334/aogh.5165 (PMC13496231; doi:10.5334/aogh.5165)
Supplement: Supplementary file 1 [file agh-92-1-5165-s1.pdf]

Supplementary File S1: Conceptual Mapping: Educational Programs and RE-AIM

RE-AIM Domain	Application to Educational Programs
**Reach**	Who participates in the training? Are learners representative of the intended workforce? What barriers affect access (e.g., time, connectivity, employment)?
**Effectiveness**	What changes occur in knowledge, skills, attitudes, confidence, or professional behavior? Are there unintended consequences?
**Adoption**	Which institutions, departments, or faculty are willing and able to deliver the program? What organizational supports are required?
**Implementation**	How is the curriculum delivered in practice? What adaptations occur? What resources, feedback mechanisms, or instructional supports are needed?
**Maintenance**	Is the program sustained over time? Is it institutionalized (e.g., credit-bearing, credentialed, embedded in curricula)? Do learner gains persist?

This alignment is especially relevant for **virtual, part-time, or LMIC training programs**, where issues of feasibility, scalability, and sustainability are central.
