# Supplementary material for: Building Health Equity Research Capacity in Myanmar Through a Virtual, Mentor-Based Training Program
Source: Ann Glob Health. 2026 Aug 18;92(1):80. doi: 10.5334/aogh.5165 (PMC13496231; doi:10.5334/aogh.5165)
Supplement: Supplementary file 2 [file agh-92-1-5165-s2.pdf]

Supplementary File S2. Student Feedback Mapped to RE-AIM Domains

RE-AIM Domain	Quantitative Indicators	Qualitative Themes (Illustrative Content)	Interpretive Contribution
**Reach**	High agreement that the virtual format, part-time structure, and scheduling enabled participation (weighted averages 1.55–1.91).	Online accessibility, outside–office-hours scheduling, and availability of training otherwise absent in Myanmar enabled participation alongside full-time employment.	Indicates that the virtual format successfully reached working professionals in a constrained context.
**Effectiveness**	Majority agreed the course was well structured and met expectations (82–91% agree/strongly agree).	Students reported gains in health equity knowledge, research design skills, and secondary data analysis; project development was described as a meaningful learning experience.	Suggests perceived educational effectiveness in building foundational knowledge and applied research competencies.
**Adoption**	Very high ratings of faculty preparedness, clarity, encouragement of participation, and willingness to recommend the course (≥90% agree/strongly agree).	Faculty expertise, responsiveness, and mentorship motivated sustained engagement.	Reflects strong learner-level adoption of the instructional and mentoring model.
**Implementation**	More neutral responses regarding workload appropriateness and Moodle usability (weighted averages ~2.3–2.45).	Students described challenges with statistical analysis, limited individualized feedback, asynchronous learning demands, and balancing coursework with professional and caregiving responsibilities.	Identifies implementation challenges related to instructional pacing, quantitative skill acquisition, and feedback intensity.
**Maintenance**	Strong willingness to recommend the course; mixed perceptions of long-term value without formal credentials.	Students emphasized the importance of certification, credit-bearing structures, continued LMS access, and potential in-person or hybrid sessions.	Highlights factors influencing perceived sustainability and long-term value beyond program completion.
