# Supplementary material for: Building Health Equity Research Capacity in Myanmar Through a Virtual, Mentor-Based Training Program
Source: Ann Glob Health. 2026 Aug 18;92(1):80. doi: 10.5334/aogh.5165 (PMC13496231; doi:10.5334/aogh.5165)
Supplement: Supplementary file 3 [file agh-92-1-5165-s3.pdf]

Supplementary File S3. Faculty Feedback Mapped to RE-AIM Domains

RE-AIM Domain	Quantitative Indicators	Qualitative Themes (Illustrative Content)	Interpretive Contribution
**Reach**	Faculty agreed that the virtual format enabled delivery of teaching despite political instability and competing professional demands.	Virtual delivery supported real-time collaboration and instructional continuity during institutional disruption.	Demonstrates feasibility of faculty participation and instructional continuity in a fragile setting.
**Effectiveness**	Weekly virtual sessions were rated as helpful for teaching course content (100% agreement).	Mentoring supported students' identification of equity-relevant variables and application of analytic concepts.	Suggests perceived instructional effectiveness in facilitating applied learning using secondary data.
**Adoption**	Most faculty reported willingness to recommend participation to colleagues.	Faculty highlighted strong coordination across lecturers and positive team-based teaching experiences.	Indicates strong faculty buy-in and willingness to re-engage despite increased instructional demands.
**Implementation**	Mixed ratings regarding student engagement in asynchronous activities and adequacy of Moodle technical support, with greater variability than for synchronous sessions.	Faculty emphasized need for more structured mentoring, improved alignment between asynchronous content and live webinars, and realistic expectations for mentoring time.	Identifies implementation challenges related to instructional integration, mentoring intensity, and differential student readiness for asynchronous learning in virtual environments.
**Maintenance**	Faculty expressed continued support for the program model contingent on refinements.	Recommendations included streamlining assignments during crisis periods, providing mentor orientation resources, and considering selective hybrid or in-person elements when feasible.	Highlights conditions required for sustainability, scalability, and long-term faculty engagement. Faculty support for sustainability was contingent on targeted refinements rather than fundamental changes to the virtual delivery model.

Note: RE-AIM domains were applied *interpretively* to organize faculty perceptions and descriptive quantitative trends rather than as formal implementation or effectiveness metrics.
