# Supplementary material for: Building Health Equity Research Capacity in Myanmar Through a Virtual, Mentor-Based Training Program
Source: Ann Glob Health. 2026 Aug 18;92(1):80. doi: 10.5334/aogh.5165 (PMC13496231; doi:10.5334/aogh.5165)
Supplement: Supplementary file 4 [file agh-92-1-5165-s4.pdf]

Supplemental File 4: Myanmar Health Equity Proposal Evaluation Rubric

Name of Student:
Project Title:
Name of Faculty

Reviewer Instructions

- Evaluate each criterion independently using the scoring guidance below.
- Assign the highest scores only to proposals that fully satisfy the criterion with minimal weaknesses.
- Deduct a small number of points for minor weaknesses and larger deductions for substantive concerns.
- Provide comments for any criterion receiving less than 70% of the available points or whenever constructive feedback would help improve the proposal.
- Base your evaluation on the quality and completeness of the proposal as submitted.

Evaluation Criterion	Max Points	Scoring Guidance	Score
1. Importance and significance of the research question	20	18–20: Addresses an important health equity problem with a compelling scientific and public health rationale.<br>14–17: Important topic with minor weaknesses in significance or justification.<br>10–13: Moderately important but limited rationale or originality.<br>0–9: Significance is unclear or poorly justified.	
2. Literature review and justification	15	14–15: Comprehensive, current, and well-synthesized literature; clearly identifies the knowledge gap and justifies the study.<br>11–13: Adequate review with minor omissions.<br>8–10: Limited review or weak linkage to the research question.<br>0–7: Inadequate review with little justification.	
3. Research objectives and research question	15	14–15: Objectives and research question are clear, specific, measurable, and logically aligned.<br>11–13: Generally clear with minor inconsistencies.<br>8–10: Objectives are somewhat vague or only partially aligned.<br>0–7: Objectives or research question are unclear, poorly formulated, or missing.	
4. Methods and scientific rigor	20	18–20: Study design, sampling, variables, data collection, analysis, and ethical considerations are appropriate, feasible, and clearly described.<br>14–17: Sound methods with several areas needing clarification.<br>10–13: Important methodological weaknesses or limited feasibility.<br>0–9: Major flaws that substantially limit the study's validity or feasibility.	
5. Potential impact and relevance to health equity	20	18–20: High potential to advance knowledge, policy, or practice and makes a strong contribution to health equity.<br>14–17: Clearly relevant with moderate potential impact.<br>10–13: Some relevance but limited impact or weak connection to health equity.<br>0–9: Minimal relevance or impact.	
6. Organization and writing quality	10	9–10: Exceptionally well organized, logical, concise, and nearly free of grammatical or formatting errors.<br>7–8: Well written with only minor issues.<br>5–6: Organization or writing occasionally impedes understanding.<br>0–4: Poor organization, numerous writing errors, or difficult to follow.	
TOTAL SCORE	100		/100
